# Evaluation of Moisture-Related Attenuation Coefficient and Water Diffusion Velocity in Human Skin Using Optical Coherence Tomography

**DOI:** 10.3390/s130404041

**Published:** 2013-03-25

**Authors:** Cheng-Kuang Lee, Meng-Tsan Tsai, Feng-Yu Chang, Chih-Hsun Yang, Su-Chin Shen, Ouyang Yuan, Chih-He Yang

**Affiliations:** 1 Department of Electrical Engineering, Chang Gung University, 259, Wen-Hwa 1st Road, Kwei-Shan, Tao-Yuan 33302, Taiwan; E-Mails: chengkuanglee@mail.cgu.edu.tw (C.-K.L.); shyanchang@hotmail.com (F.-Y.C.); oyy@mail.cgu.edu.tw (O.Y.); orton.yang@gigabyte.com (C.-H.Y.); 2 Graduate Institute of Medical Mechatronics, Chang Gung University, 259, Wen-Hwa 1st Road, Kwei-Shan, Tao-Yuan 33302, Taiwan; 3 Department of Dermatology, Chang Gung Memorial Hospital, 5 Fusing Street, Kwei-Shan, Tao-Yaun 33302, Taiwan; E-Mail: dermadr@hotmail.com; 4 Department of Ophthalmology, Chang Gung Memorial Hospital, 5 Fusing Street, Kwei-Shan, Tao-Yaun 33302, Taiwan; E-Mail: suchin@adm.cgmh.org.tw

**Keywords:** optical coherence tomography, moisture, attenuation coefficient, water diffusion

## Abstract

In this study, time-resolved optical coherence tomography (OCT) scanning images of the process of water diffusion in the skin that illustrate the enhancement in the backscattered intensities due to the increased water concentration are presented. In our experiments, the water concentration in the skin was increased by soaking the hand in water, and the same region of the skin was scanned and measured with the OCT system and a commercial moisture monitor every three minutes. To quantitatively analyze the moisture-related optical properties and the velocity of water diffusion in human skin, the attenuation coefficients of the skin, including the epidermis and dermis layers, were evaluated. Furthermore, the evaluated attenuation coefficients were compared with the measurements made using the commercial moisture monitor. The results demonstrate that the attenuation coefficient increases as the water concentration increases. Furthermore, by evaluating the positions of center-of mass of the backscattered intensities from OCT images, the diffusion velocity can be estimated. In contrast to the commercial moisture monitor, OCT can provide three-dimensional structural images of the skin and characterize its optical property, which together can be used to observe morphological changes and quantitatively evaluate the moisture-related attenuation coefficients in different skin layers.

## 1. Introduction

Skin is the physical barrier for the human body, tasked with preventing damage from various external stimuli and preventing the loss of water [1]. Additionally, skin's softness is related to the moisture in the skin, which is essential for protecting the body. It is composed of three layers: the epidermis (EP), the dermis (DM), and the subcutaneous layer. The EP layer is the outermost layer and acts as a protective barrier. The stratum corneum (SC) is the outer layer of epidermis and is composed of dead skin cells made of keratin. Additionally, water in the skin plays an important role in gland secretions, regulation of body temperature, and the prevention of aging. Many approaches for measuring water concentration in human skin have been proposed [2–4], including electric conductance [5], transepidermal water loss [6], Fourier transform infrared spectroscopy [7], photothermal imaging [8], and confocal Raman spectroscopy [9]. However, the proposed approaches are limited to measuring the water concentration in the SC layer, and such information is not enough to completely characterize the skin's properties.

Over the last few decades, optical sensing and imaging have attracted much attention in biomedical applications such as near-infrared spectroscopy [10], photoacoustic microscopy [11,12], nonlinear microscopy [13,14], and optical coherence tomography (OCT) [15,16]. Compared with other optical imaging techniques, OCT has the advantages of deeper imaging depth, requiring no contrast agents, and high imaging speed. Based on the interferometer configuration, either two-dimensional or three-dimensional micro-structural information can be reconstructed without destroying the sample. Since 1991, many research groups have demonstrated that OCT can be applied in various biomedical fields such as ophthalmology, dermatology, and oncology [17–19]. In the last decade, the imaging speed and system sensitivity have been greatly improved due to the development of Fourier-domain OCT (FD-OCT) without mechanical scanning in the reference arm of the interferometer. Furthermore, FD-OCT includes two different configurations known as swept-source OCT (SS-OCT) [20–22] and spectral-domain OCT (SD-OCT) [23–25]. Aside from obtaining structural information, OCT can perform functional imaging including tissue birefringence, blood flow velocity and angiography [26–28].

Many dermatological studies using OCT have been reported [29–35], most of which focus on the detection of pathological changes in the skin due to skin disorders. Additionally, dermal birefringence, which can be utilized for the diagnosis of sun damage [33] or for the determination of burn depth [34], can be visualized using polarization-sensitive optical coherence tomography (PS-OCT). Furthermore, Yasuno *et al.* were able to differentiate young and old photo-aged human skin based on a birefringence analysis using PS-OCT [35]. In addition to characterizing skin morphology, OCT has been proposed by Ohmi *et al.* as a tool for performing dynamic analysis of mental sweating from human fingertips [36]. The same group was also able to visualize the dynamics of the small arteries and veins of human fingers using OCT [37].

In this study, an SS-OCT system is implemented for the investigation of moisture-related optical property of human skin. In our experiments, OCT scans taken every 3 min after soaking the palm in water were used to observe water diffusion and evaluate the moisture-related attenuation coefficient of human skin. The time-resolved OCT scans revealed the process of water diffusion in the skin, which we then analyzed quantitatively along with the skin's moisture by evaluating the skin's attenuation coefficients. Then, the OCT scanning results were compared with the measurements made by a commercial moisture monitor. Furthermore, to investigate the diffusion velocity in skin, the positions of center-of-mass of backscattered intensities in the longitudinal direction from OCT images are evaluated.

## 2. Experimental Section

**Figure 1.**
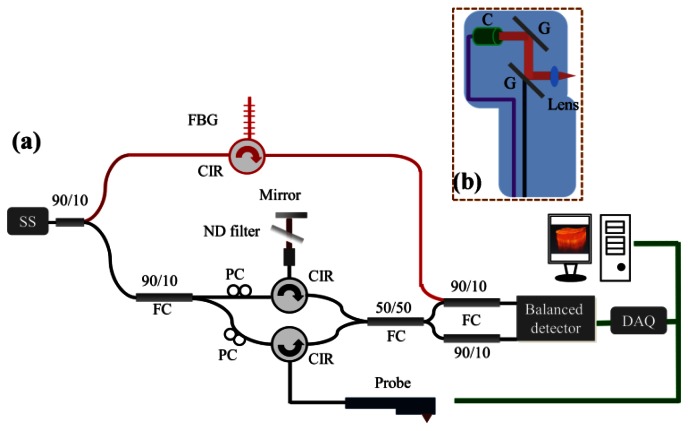
(**a**) Schematic diagram of the portable SS-OCT system used for studying water diffusion in the skin. (**b**) Layout of the handheld probe. PC: polarization controller, CIR: optical circulator, FBG: fiber Bragg grating, FC: fiber coupler, DAQ: data acquisition board, C: collimator, and G: galvanometer.

Figure 1(a) shows a schematic diagram of the portable SS-OCT system used for studying water diffusion in the skin [38]. The central wavelength and the scanning range of the swept source are 1,310 nm and 110 nm, respectively. This source can provide an output power of 6 mW and a sweeping rate of 30 kHz. It is connected to a Mach-Zehnder interferometer, consisting of two circulators and two couplers. Ten percent of the output power from the swept source is connected to a narrowband fiber Bragg grating (FBG) to generate an A-scan trigger for each A-scan. The narrowband FBG has a Bragg wavelength of 1,275 nm, and the reflected signal from the FBG is combined with the interfered signal by a 10/90 fiber coupler. To eliminate the DC component of the interfered signal, another 10/90 fiber coupler is used before the balanced detector (PDB150C, Thorlabs). Finally, the data from the balanced detector is sampled with a high-speed digitizer at a sampling rate of 100 MB/s (PXIe-5122, National Instruments). Based on this mechanism, the time-induced phase errors can be greatly reduced, and only half the on-board memory of the digitizer is required for data acquisition. In the sample arm, a palm-held probe is implemented for skin scanning. Figure 1(b) shows the layout of the probe for scanning human skin. A single-mode fiber with an FC/APC connector is connected to a collimator, and the output light beam was incident onto a two-axis galvanometer, which provides lateral and transverse scanning. The light beam is focused by an achromatic lens having a focal length of 10 mm, resulting in the focusing of the light beam at a depth of 300 μm beneath the sample surface. In this OCT system, the frame rate can achieve 50 frames per second, each consisting of 600 A-scans.

Water concentration in the skin is an important factor in preventing skin damage from external infections and aging. To increase the water concentration in skin, the left palm of a 23-year-old volunteer was soaked in water. Because lipids on the SC influence water diffusion and hydration, the volunteer washed his palm with soap to speed up water diffusion before the measurement. The index fingertip was scanned using the OCT system at 0, 3, 6, 9, 12, 15, 18, and 30 min after soaking. After each OCT scan, a commercial moisture monitor (ZRH-009, Chung Yun Industrial) that assesses moisture levels based on the electrical conductance measurement was also used to measure the water concentration. To facilitate scanning of the same region of the index fingertip in each measurement, the scanned region was marked. However, the regions scanned in each measurement were not exactly identical, even with the marking, although each scan did cover most of the marked region. After each measurement, the palm was kept soaked in water. To obtain the statistical results, the experiment was repeated for seven times. The water concentration of the skin increases as the immersion time increases due to water diffusion.

## 3. Results and Discussion

### 3.1. Evaluation of Moisture-Related Attenuation Coefficient

**Figure 2.**
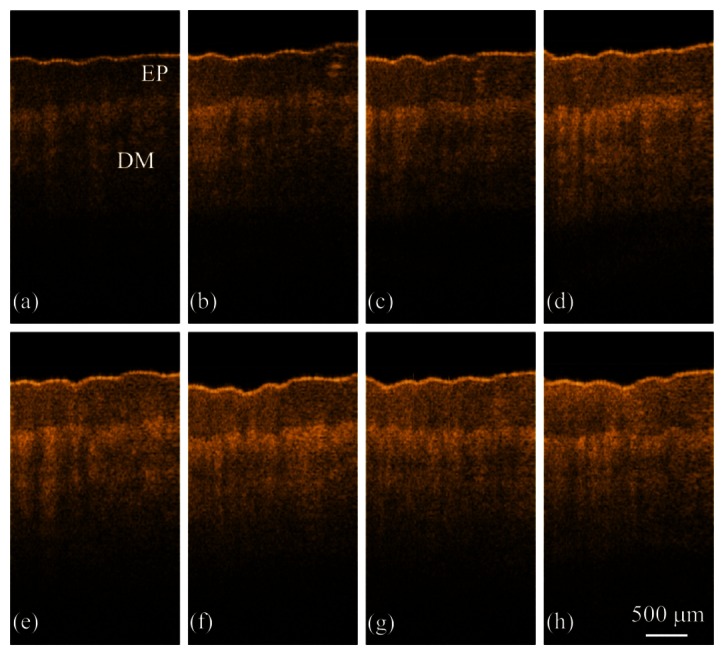
*In vivo* OCT scanning results of the left index finger obtained at 0 (**a**); 3 (**b**); 6 (**c**); 9 (**d**); 12 (**e**); 15 (**f**); 18 (**g**); and 30 min (**h**) after soaking the left palm in water. Each OCT image consists of 600 A-scans.

To clearly illustrate the differences in the OCT images induced by the different water concentrations, we present here the two-dimensional OCT images of the fingertip. Figure 2 shows *in vivo* OCT scanning results of the left index finger obtained at 0 (a), 3 (b), 6 (c), 9 (d), 12 (e), 15 (f), 18 (g), and 30 min (h) after soaking the left palm in water. From the images, different layers of the skin, including the EP, and DM layers, can be identified. From Figure 2, one can see that the backscattered intensity at greater depths increases as the immersion time increases.

To quantitatively evaluate the changes in OCT intensity during water diffusion, the attenuation coefficients of the EP and DM layers can be evaluated based on the Beer-Lambert law. Thus, the OCT intensity can be expressed as:

(1) $$I(z)\alpha \sqrt{\exp(-2\mu_{t}z)}/\sqrt{1+{\left(\frac{z-z_{cf}}{Z_{R}}\right)}^{2}}$$

where *μ_t_* is the attenuation coefficient, *z_cf_* is the depth of the focal point, and *z_R_* is the Rayleigh length. The numerator of the right side of Equation (1) describes the intensity decay induced by the sample, and the denominator results from the effect of the confocal point spread function of the focusing lens. When the sample is scanned with our OCT system, the sample directly contacts the probe, making the focal point approximately fixed at a depth of 300 μm beneath the sample surface. Additionally, the Rayleigh length of the focusing lens is determined to be approximately 239 μm. As the value of the denominator in Equation (1) ranges between 1.6 and 3.9, the effect of the confocal point spread function was ignored in this study. Hence, Equation (1) can be simplified to be:

(2) $$I(z)\alpha \exp(-\mu_{t}z)$$

Therefore, the attenuation coefficients of skin including EP and DM layers can be evaluated from OCT intensities, as shown in Equation (2).

**Figure 3.**
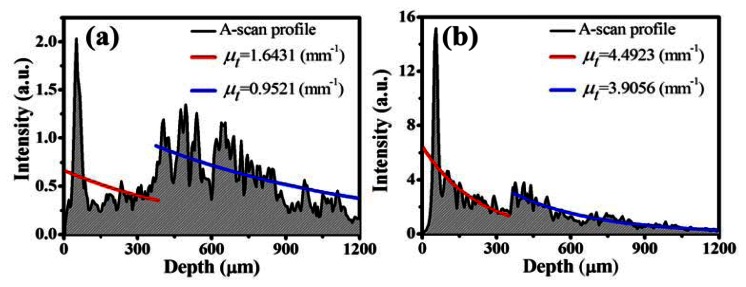
A-scan profiles averaged over 33 μm in lateral distance and the evaluated attenuation coefficients in the EP and DM layers of Figure 2(a) and 2(h), respectively.

To evaluate the attenuation coefficient, we averaged the 11 adjacent A-scan profiles in this study, which correspond to 33 μm in the lateral distance. Figure 3(a,b) shows the A-scan profiles of Figure 2(a) and 2(h), which were acquired by averaging the 295th to the 305th A-scan profiles of Figure 2(a) and 2(h), respectively.

To evaluate the attenuation coefficients of EP and DM layers, a segmentation algorithm was applied to obtain the EP and DM layers [39]. In Figure 3(a,b), the red and blue lines represent the exponential decay fits of the EP and DM layers, respectively. In Figure 3(a), the evaluated *μ_t_* values of the EP and DM layers are 1.6431 mm^−1^ and 0.9521 mm^−1^, respectively. After soaking the left palm in water for 30 min, the evaluated *μ_t_* values of the EP and DM layers become 4.4923 mm^−1^ and 3.9056 mm^−1^, respectively. From these results, one can see that the *μ_t_* values of the EP and DM layers in Figure 3(b) are greater than those of Figure 3(a), resulting from the enhanced backscattered intensity due to water diffusion into the skin.

**Figure 4.**
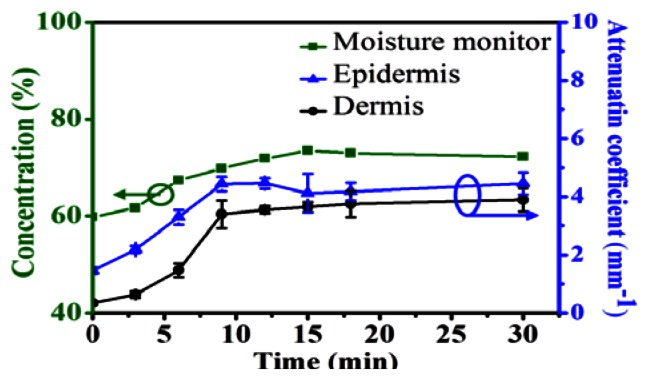
The evaluated scattering coefficients of the EP and DM layers of Figure 2(a–h), along with the measured results from the commercial moisture monitor.

To statistically analyze the relationship between the moisture and the attenuation coefficient, the attenuation coefficients of the EP and DM layers of Figure 2(a–h) were evaluated and are shown in Figure 4, which also displays the measured results from the commercial moisture monitor as a green line. In Figure 4, the averaged attenuation coefficients with standard deviations are obtained over seven measurements. From the results, the evaluated attenuation coefficients based on the OCT data and the measured results from the commercial moisture monitor show the same trend as a function of time. However, only the moisture of the SC layer can be measured by the commercial moisture monitor. Compared with the commercial product, OCT can evaluate the attenuation coefficients of the EP and DM layers, which are moisture-related. From the evaluated *μ_t_* values, the water concentration becomes saturated in the EP and DM layers after soaking the palm in water for 18 min, a result mirrored by the moisture monitor data.

### 3.2. Estimation of Diffusion Velocity in Skin

**Figure 5.**
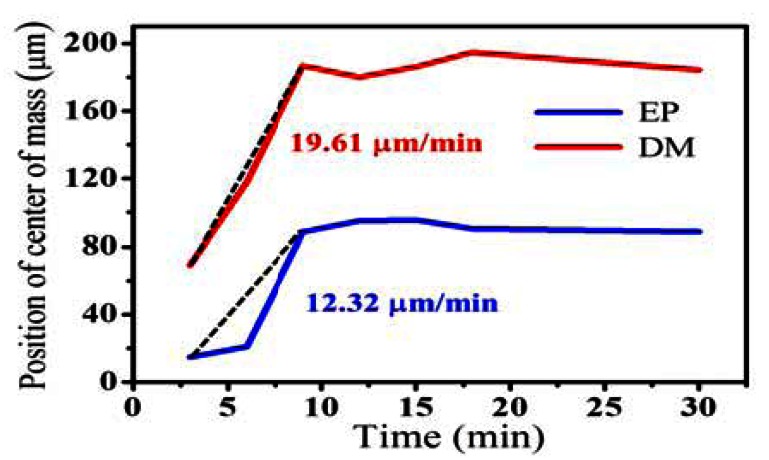
The evaluated *CM* values of the EP and DM layers from Figure 2(a–h).

Moisture is an important issue to maintain skin's softness to protect the body, and water in the skin can regulate the body temperature and prevent from aging. Thus, water loss from skin or water diffusion into skin plays an important role. To further evaluate the water diffusion velocity in the skin, we propose to estimate the positions of center-of-mass of backscattered intensities from OCT images. As abovementioned, the attenuation coefficients in the EP and LP layers are related to the water concentration in skin, referring to the changes in the backscattered intensities of OCT images. To evaluate the enhancement of backscattered intensities due to the increased water concentration, we evaluate the intensity variance at each image pixel of every two successive images in Figure 2. However, because the thicknesses of the EP and DM layers in Figure 2(a–h) are not uniform, an edge-detection algorithm is applied to segment the two layers. Thus, based on the edge-detection algorithm, the two boundaries including the skin surface and the basement membrane (the boundary between the EP and DM layers) can be obtained. Subsequently, in the EP layer, the thickness of 300 μm beneath the skin surface and the thickness of 500 μm beneath the basement membrane in the DM layer are used for the evaluation of intensity variance between two successive images. Therefore, seven intensity variance images can be obtained from Figure 2(a–h). Then, the position of center-of-mass (*CM*) of each A-scan in an OCT intensity variance image can be evaluated from each intensity variance image, which can be expressed as:

(3) $$CM=\sum_{j=1}^{n}\left[\sum_{i=1}^{m}I_{j}(z_{i})\cdot z_{i}/\frac{1}{m}\cdot \sum_{i=1}^{m}I_{j}(z_{i})\right]$$

where *I_j_(z)* is the intensity profile of the *j*th A-scan as a function of depth, *z*, in the intensity variance image. *n* represents the selected adjacent A-scan number for obtaining a averaged *CM* value, and *m* is the selected pixels in the depth range of the EP or DM layer for the evaluation of the *CM* value, corresponding to z*_m_* to be the total selected range. In this study, the selected depth ranges of EP and DM layers are chosen to be 300 μm starting from the skin surface and 500 μm starting from EP/DM boundary, respectively. Furthermore, *n* is equal to 11, corresponding to an averaged transverse range of 33 μm. Then, the *CM* values estimated from Figure 2 are shown in Figure 5. In Figure 5, the red and blue lines represent the *CM* values as a function of time, respectively. From the results, the *CM* values increase obviously before the 9th minutes and become slightly changed during the 9th to 18th minutes. After soaking the hand in water for 18 minutes, the CM values become approximately constant, referring to the saturation of water concentration in the skin. Furthermore, according to the movement of *CM* position, the diffusion velocities of the EP and DM layers can be obtained to be 12.32 μm/min and 19.61 μm/min, as shown in Figure 5. Such indicator can be useful to evaluate the skin conditions in clinical.

## 4. Conclusions

In summary, we have used OCT to make time-resolved measurements of water diffusion in the skin. In our experiments, the palm was immersed in water, and the skin was scanned with OCT every 3 min. The backscattered intensities in the OCT images are enhanced due to water diffusion in the skin. To quantitatively evaluate the water diffusion and concentration, the moisture-related attenuation coefficient can be used. A comparison of the attenuation coefficients of the epidermis and dermis layers before and after soaking the palm in water for 30 min revealed that the attenuation coefficients of the epidermis and dermis layers increase after soaking the palm in water. Additionally, we analyzed the moisture-related attenuation coefficients as a function of increasing immersion time and compared the OCT results with the measured moisture from the commercial moisture monitor. The moisture-related attenuation coefficients evaluated using OCT show the same trend as the results from the commercial product. Furthermore, the diffusion velocity of water in human skin can be estimated by evaluating the positions of center-of-mass of intensity variance OCT images, obtained from the successive OCT images. From the results, the water diffusion velocities in the EP and DM layers of one volunteer' skin are 12.32 μm/min and 19.61 μm/min, respectively. However, such information still cannot be obtained from any other commercial products.
